# Molecular detection of *Chlamydia abortus* in endometrial biopsies of mares from western Canada

**DOI:** 10.1177/10406387241267864

**Published:** 2024-08-17

**Authors:** R. Madison Ricard, Bruce Wobeser

**Affiliations:** Department of Veterinary Pathology, Western College of Veterinary Medicine, University of Saskatchewan, Saskatoon, SK, Canada

**Keywords:** Canada, *Chlamydia*, *Chlamydia abortus*, endometrial, horses

## Abstract

*Chlamydia abortus* is a reported cause of infertility and endometritis in sheep, cattle, and pigs; however, the association between uterine disease and *C. abortus* is poorly understood in horses. Recently, a high prevalence of *C. abortus* in equine aborted chorioallantoises was reported in horses in western Canada. Based on this high prevalence, investigation into the effects of *C. abortus* on infertility and endometritis in western Canadian mares is prudent. We examined 98 formalin-fixed, paraffin-embedded endometrial biopsies from western Canada submitted between 2014 and 2022 using a *Chlamydia*-specific 16S rRNA PCR test; 40 samples tested positive for *Chlamydia* on PCR, and 28 were sequenced as *C. abortus*. The *C. abortus*–positive cases were primarily associated with a history of failure to conceive, early embryonic loss, or abortion. Our findings suggest that *C. abortus* may be a cause of conception failure and abortion in horses in western Canada.

*Chlamydia abortus* is a gram-negative bacterium that is the causative agent of enzootic ovine abortion.^13^ In horses, *C. abortus* has been historically associated with only occasional cases of abortion.^1,4^ In a 2023 investigation into cases of equine abortion in western Canada, 26 of 99 abortion cases were positive on *Chlamydia* PCR testing, and, in 22 of the cases, *C. abortus* was subsequently identified based on DNA sequencing.^12^ The findings in western Canada were unexpectedly high compared to similar studies from Europe, suggesting the need for further investigation.^12^ To date, the relationship between *C. abortus* and equine abortion has not been fully established,^10,12^ as identification of the pathogen on PCR testing alone does not necessarily implicate it as the abortigenic agent.

In cattle and pigs, *C. abortus* has been identified as an otherwise subclinical cause of infertility, with rare cases of more severe disease resulting in abortion.^3,6,8,11^ In sheep, there is also evidence of *C. abortus* causing infertility, despite the pathogen typically being associated with abortion.^9^ PCR is the most commonly used method for the detection of *C. abortus* in these species, as bacterial culture requires specialized techniques that are not widely available and require live organisms for detection.^6,11,13^

A 2019 study from Italy identified *C. abortus* in cytobrush samples from the uterus of 6 mares with a history of infertility, embryonic resorption, and abortion.^10^ Four of the mares were treated with oxytetracycline intrauterine infusions, and subsequently conceived after testing negative on a repeat *Chlamydia* PCR test.^10^ These findings suggest that *C. abortus* infection may contribute to infertility and abortion in mares, similar to other species.^10^

Based on the findings in Italy, and the high prevalence of *C. abortus* identified in abortion cases in western Canada, investigation into the role of *C. abortus* in equine infertility in western Canada is prudent. We investigated whether *C. abortus* is present in endometrial biopsies of mares from an equine population similar to that in the 2023 equine abortion investigation.^12^ We expected that *C. abortus* would be identified in the tested endometrial biopsies and that most of the positive cases identified would be associated with a history of infertility or abortion. Positive cases were also expected to have mild histologic endometritis, similar to the Italian study.^10^

Equine endometrial biopsies submitted between 2014 and 2022 were selected from the database of Prairie Diagnostic Services (PDS; Saskatoon, Saskatchewan, Canada) by searching for keywords “endometrial” and “Kenney-Doig”. This search query yielded 243 endometrial biopsy submissions. Each submission was reviewed, and 25 cases that did not have a supplied history were removed from the study group. We randomly selected 100 submissions by simple random sampling by random number attribution from the remaining submissions pool. The formalin-fixed, paraffin-embedded (FFPE) tissue block for each submission was retrieved from the PDS archive.

For each block, DNA retrieval was performed using methods described previously.^12^ Five 20-µm microtome shavings were taken sequentially from each block and deparaffinized. DNA extraction was performed by adding 100 mM NaCl, 500 nM Tris, and 10% sodium dodecyl sulfate lysis buffer solution and 5 µL of proteinase K to each sample and incubating the samples overnight at 56°C. Phenol–chloroform–isoamyl alcohol was used to remove the proteins, allowing the remaining DNA to be precipitated in 2 volumes of 100% ethanol and 0.1 volumes of 3 M sodium acetate. The DNA was resuspended in 50 µL of Tris–EDTA buffer for reading on a spectrophotometer (Nano Drop 2000C; Thermo Scientific) to ensure adequate nucleic acid concentrations. Prior to performing the *Chlamydia* PCR test, each sample underwent PCR with a primer for the beta-actin reference gene, as described previously, to confirm the presence of amplifiable DNA.^12^

We performed our *Chlamydia* PCR assay using primers 16SIGF/16SIGR (16SIGF: 5′-GATGAGGCATGCAAGTCGAACG-3′; 16SIGR: 5′-CCAGTGTTGGCGGTCAATCTCTC-3′).^2^ The primers produced a 278-bp fragment of a conserved region of the 16S rRNA gene. The PCR reaction utilized 2 µL of sample DNA with 3.5 mM MgCl₂, 2 µL of 0.4 mM of deoxyribonucleotides (dNTP; Thermo Scientific), 5 µL of each 1.0 µM forward (16SIGF) and reverse (16SIGR) primer (Sigma), 2 U DNA polymerase (Quantabio), and distilled water added to bring the total volume to 50 µL. PCR cycling (Mastercycler; Eppendorf) involved an initial denaturation step of 15 min at 95°C, followed by 45 cycles of denaturation at 94°C for 30 s, primer annealing at 70°C for 30 s, and extension at 72°C for 45 s, with a final extension step of 5 min at 72°C.

The amplification products were run on 1.2% Tris–EDTA agarose gel containing ethidium bromide. Each set of samples for electrophoresis included a blank wax negative control, blank extraction negative control, and no-template controls. PCR-confirmed cases of sheep abortions were included as positive controls. The agarose gels were examined under UV light for the presence of bands, with bands at ~278 bp being identified as *Chlamydia*-positive cases. Test-positive samples were purified with a PCR purification kit (Qiagen) and sequenced (Macrogen). Each sequence was compared to bacterial sequences in GenBank using BLAST (https://blast.ncbi.nlm.nih.gov/Blast.cgi), with the BLAST result with the smallest E value identified as the most likely bacterial species for that case.

Based on the history provided with the endometrial biopsy submission, the study population cases were categorized based on province of origin, breed of the mare, age of the mare, parity of the mare, reason for submitting the biopsy, whether there was a reported history of abortion or early embryonic loss, and Kenney–Doig score. Categories for dam breed included baroque, light pleasure, draft, stock horse, Thoroughbred, Warmblood, pony, and other. Dam age was categorized into 4 ranges: young mares <8-y-old, mature mares 8–13-y-old, aging mares 14–18-y-old, and senior mares ≥19-y-old. Dam parity was a binary between maiden mares who had not had previous foals, and multiparous mares. Reason for biopsy was based on interpretation of the history submitted, and was categorized into failure to conceive, history of abortion or early embryonic loss, pre-breeding examination, and other. The Kenney–Doig score was I–III, per the originally proposed rating system.^7^

Ninety-eight of the 100 randomly selected submissions had amplifiable DNA present using beta-actin reference gene conventional PCR primers. Of the 98 samples, 40 were positive for *Chlamydia* species on conventional PCR. All 4 western Canadian provinces (British Columbia, Alberta, Saskatchewan, Manitoba) that submit to PDS had positive cases identified. No positive cases were identified from the seven 2014 submissions tested. However, positive cases were identified in every subsequent year, up to 2022.

For the 40 *Chlamydia*-positive samples, the biopsies were primarily submitted due to a history of abortion or early embryonic loss (*n* = 12) or failure to conceive (*n* = 21). One case was submitted because of a history of multiple weak-born foals from the same mare, and 1 case had a history of unexplained vaginal discharge; 5 cases were submitted as part of a routine pre-breeding examination. With a range of I–III, most submissions received a Kenney–Doig grade of IIA (*n* = 14) or IIB (*n* = 18).

In comparison, the 58 test-negative samples had 35 cases of failure to conceive, 9 cases with an abortion or early embryonic loss history, 13 pre-breeding examinations, and 1 mare with a known history of endometritis. Test-positive cases did not have a statistically significant difference in the distribution of these categories compared to test-negative cases on a Fisher exact test (*p* = 0.186). Similarly, most negative cases received a Kenney–Doig score of IIA (*n* = 17) or IIB (*n* = 29). There was no significant difference between Kenney–Doig scores for positive versus negative cases on a Fisher exact test (*p* = 0.862). There were also no statistically significant differences between positive and negative cases for breed category (*p* = 0.494), age group of the mare (*p* = 0.231), or parity of the mare (*p* = 1.000).

Sequencing of the 40 test-positive cases identified 28 cases of *C. abortus* with over 95% identity (accession NZ_LS450958.2). Nine of the remaining cases had a low identity percentage (<95%), and were considered *Chlamydia*-like species. Three cases could not be identified due to sequencing failure (Table 1).

**Table 1.** Reasons for submission for 40 equine endometrial biopsies that tested positive for *Chlamydia* sp. on conventional PCR, and distribution of *Chlamydia* sp. identified and Kenney–Doig scale ratings for each reason for submission.

Reason for submission	No. of PCR-positive submissions	*Chlamydia* sp. distribution	Kenney–Doig scale distribution
Failure to conceive	21	16 *C. abortus*; 4 *Chlamydia*-like species; 1 sequencing failure	1 grade I 7 grade IIA 9 grade IIB 4 grade III
History of abortion or early embryonic loss	12	8 *C. abortus*; 2 *Chlamydia*-like species; 2 sequencing failures	1 grade I 5 grade IIA 5 grade IIB 1 grade III
Pre-breeding examination	5	4 *C. abortus*; 1 *Chlamydia*-like species	1 grade I 1 grade IIA 3 grade IIB
Other*	2	2 *Chlamydia*-like species	1 grade IIA 1 grade IIB

* One case was submitted due to a history of weak-born foals, and one case was submitted due to a history of unexplained vaginal discharge.

Our finding of 40 of 98 samples test-positive for *Chlamydia* PCR parallels findings of *Chlamydia* in equine aborted chorioallantoises,^12^ and suggests that *C. abortus* may be a notable pathogen of the equine reproductive tract in western Canada. Many of the test-positive cases had a history of abortion, early embryonic loss, or failure to conceive, similar to the other findings.^10^ In that 2019 study, the researchers were able to treat *C. abortus*–positive mares, allowing the mares to conceive during the same breeding season.^10^ Therefore, it seems likely that affected western Canadian mares may be treatable. Developing a treatment protocol for *Chlamydia*-positive mares has the potential to improve the notably high rate of embryonic loss and infertility experienced by the equine breeding population.^5^

Unlike the 2019 study,^10^ which noted primarily mild endometritis, most of our cases received a moderate endometritis score on the Kenney–Doig scale. The reason for the differences between the 2 studies is unknown; however, it may be related to known weaknesses of the Kenney–Doig scale that may encourage pathologists to select middle-ranked values.^14^ Therefore, it is unknown how the test-positive cases in our study truly compare to the 2019 study^10^ regarding the severity of endometritis.

One interesting test-positive case was a mare with a history of multiple weak-born foals. Although this case sequenced as a *Chlamydia*-like species, rather than *C. abortus*, weak-born neonates from *C. abortus* infection has been described in pigs,^8^ cattle,^2^ and sheep.^9^ Long-term effects of subclinical *C. abortus* infection has also been described in calves 2–7-mo-old, in which subclinical carriers had a lower body weight and leukopenia relative to non-carrier calves.^11^ The association of our case with *Chlamydia* species suggests that further investigation into the role of *Chlamydia* in the neonatal health of foals may be prudent.

One major limitation of our study is that we included only mares with endometrial biopsies taken for reproductive purposes. This sampling may bias toward higher value mares, whose owners have a vested economic interest in the mare’s reproductive health. The sampling is also limited to mares who are actively being bred in most cases, rather than a representation of the general mare population. In many cases, endometrial biopsies are only performed on mares with a previous history of reproductive failure, which further biases the sample. In our dataset, only 18 of the 98 cases were submitted as part of a pre-breeding examination, reflecting this bias. The effect of this sampling bias on our findings is unknown.

Using FFPE tissues may also lead to underreporting of PCR-positivity, as formalin fixation can disrupt DNA integrity. A short primer was used for our PCR protocol to maximize primer annealing; however, it is possible that some DNA fragments relevant to our study were too small to anneal to our primers, resulting in underreporting. Additionally, conventional PCR as a detection method does not allow identification of the histologic location of *C. abortus*, necessitating further investigation using modalities such as immunohistochemistry. Finally, sequencing failure of 3 of the 40 samples submitted for sequencing prevented identification of the *Chlamydia* species for these samples. However, the small number of failed samples is unlikely to significantly impact the results of our study.

*C. abortus* is a potentially zoonotic pathogen that can cause flu-like symptoms and atypical pneumonia in people.^13^ The pathogen is particularly significant in pregnant women, causing abortion and placental dysfunction.^13^ Most cases develop after exposure to contaminated fetal membranes and fluids during lambing.^13^ Our investigation suggests that mares may potentially carry *C. abortus*, similar to sheep and cattle. Based on these findings, we recommend that pregnant women avoid contact with equine fetal membranes and that non-pregnant individuals handling fetal membranes wear personal protective equipment, such as gloves.^13^
